# Supplementary material for: Identification and Quantification of Bioactive Compounds in Organic and Conventional Edible Pansy Flowers (Viola × wittrockiana) and Their Antioxidant Activity
Source: Plants (Basel). 2023 Mar 10;12(6):1264. doi: 10.3390/plants12061264 (PMC10057616; doi:10.3390/plants12061264)
Supplement: Supplementary file 1 [file plants-12-01264-s001.zip › plants-2268925-supplementary.pdf]

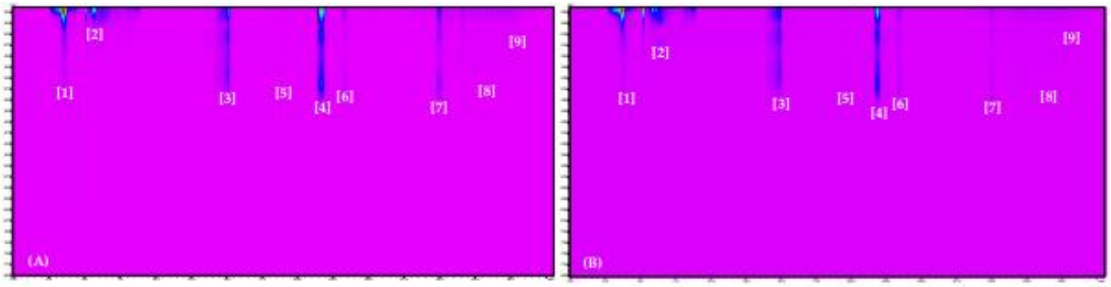

Figure S1. The pictures of HPLC-DAD spectra of identified polyphenols in organic (A) and conventional (B) pansy flowers. [1] galic acid, [2] chlorogenic acid, [3] caffeic acid, [4] p-coumaric acid, [5] quercetin-3-O-rutinoside, [6] myrycetin, [7] quercetin, [8] quercetin-3-O-glucoside, [9] kaempferol.

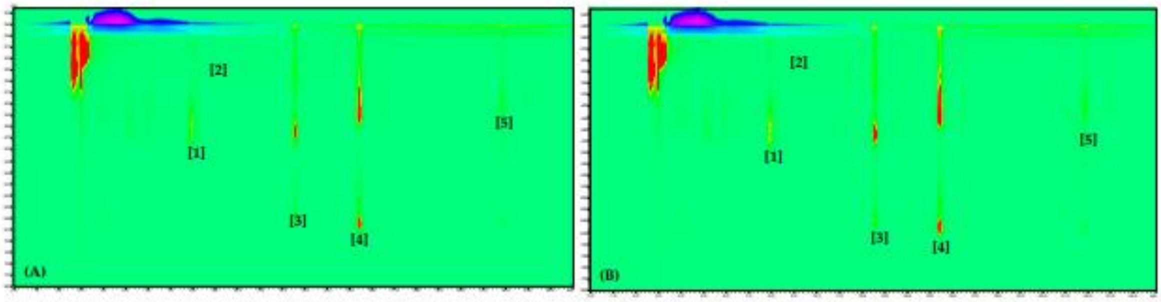

Figure S2. The pictures of HPLC-DAD spectra of identified carotenoids and chlorophylls in organic (A) and conventional (B) pansy flowers. [1] lutein, [2] zeaxanthin, [3] chlorophyll b, [4] chlorophyll a, [5] beta-carotene

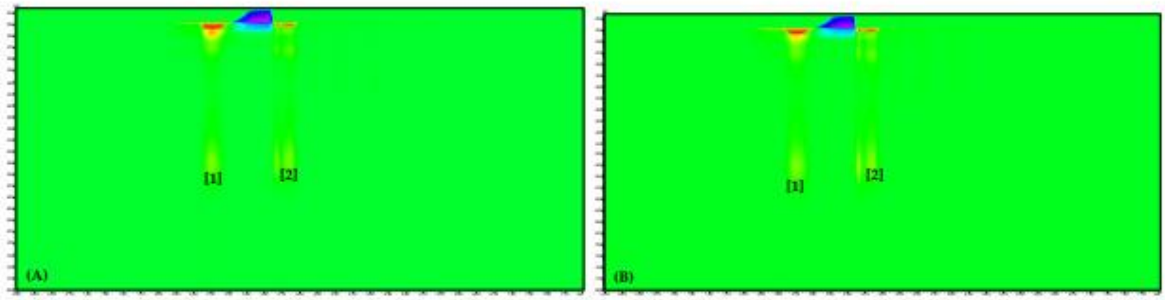

Figure S3. The pictures of HPLC-DAD spectra of identified anthocyanins in organic (A) and conventional (B) pansy flowers. [1] cyanidin-3-O-rutinoside, [2] cyanidin-3-O-glucoside

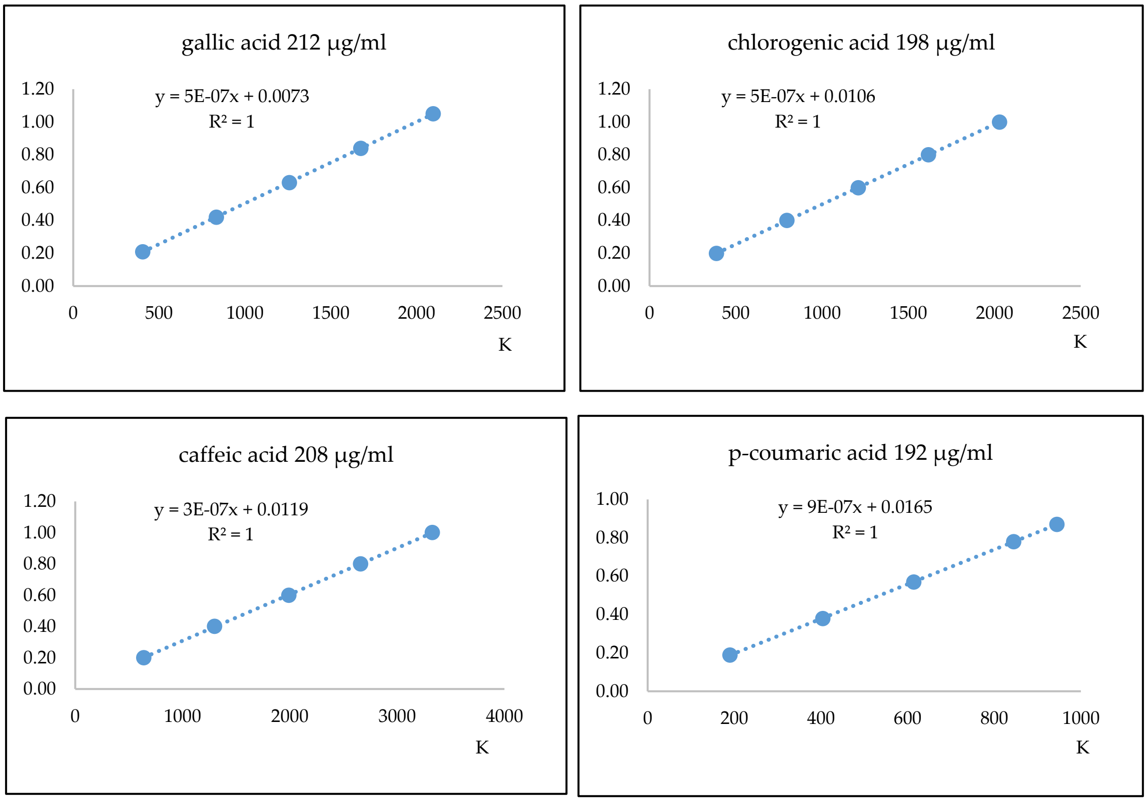

Figure S4 Standard curves for phenolic acids identified in pansy flowers petals

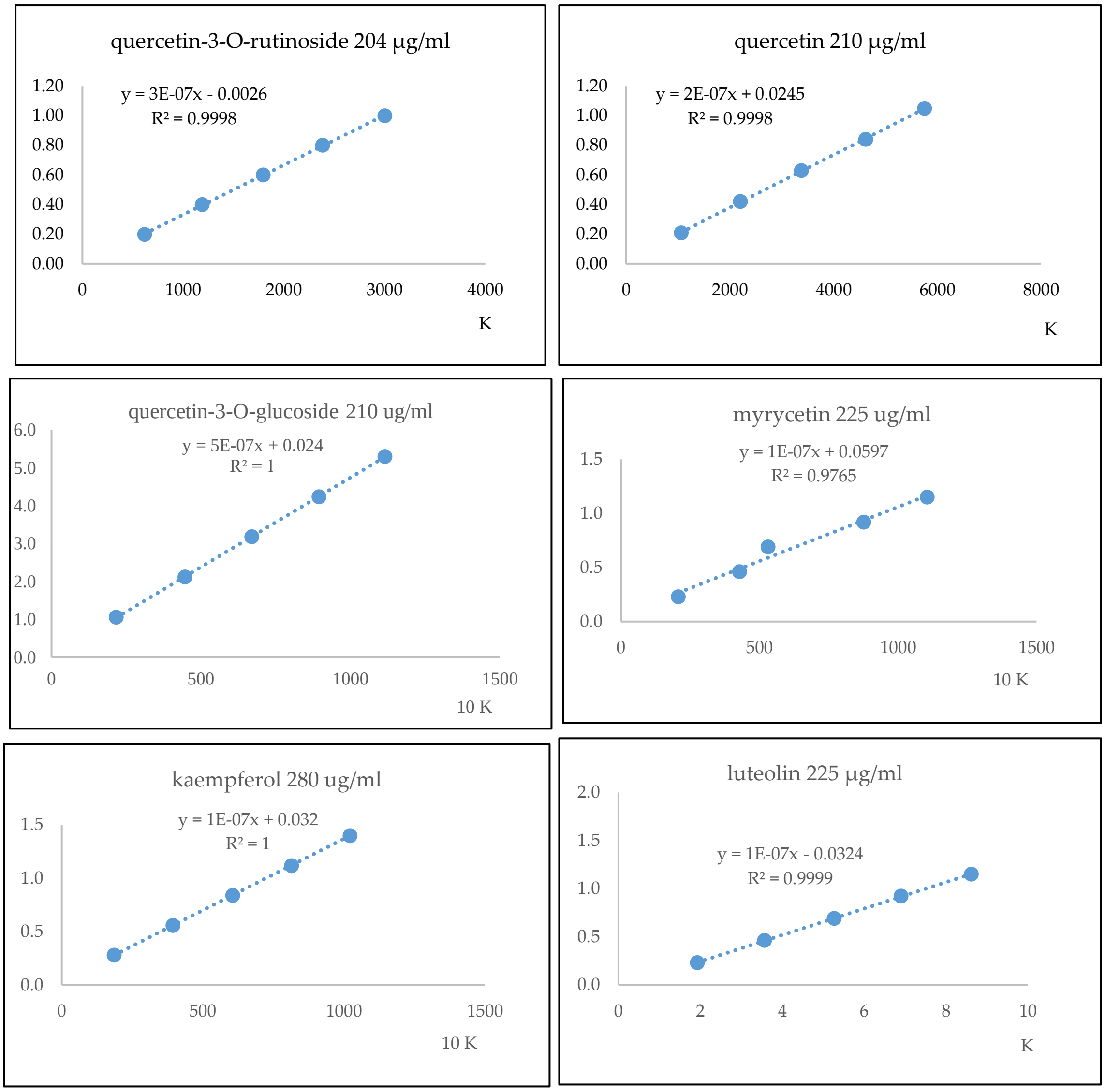

Figure S5 Standard curves for flavonoids identified in pansy flowers petals

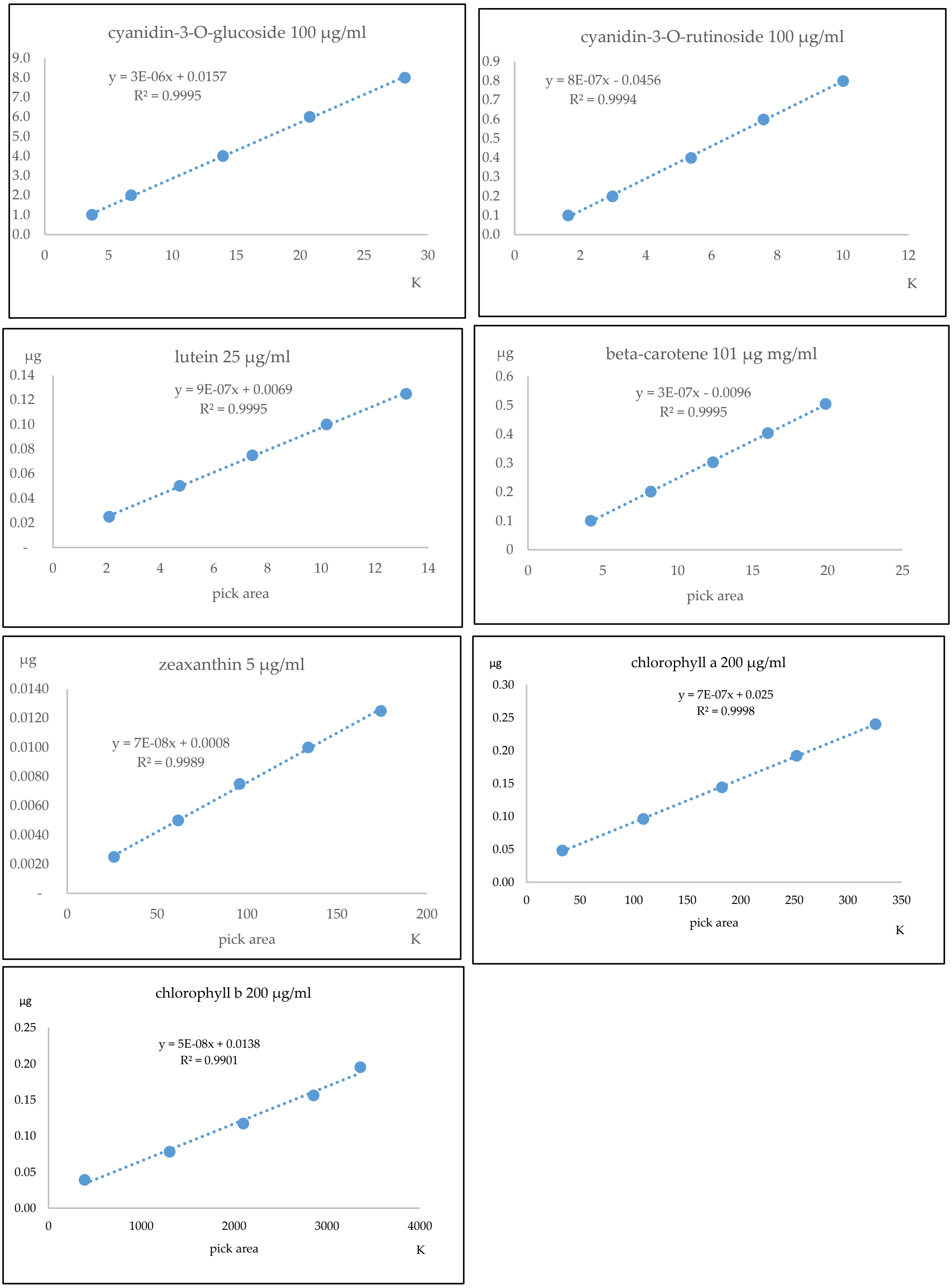

Figure S6 Standard curves for colorants (carotenoids, anthocyanins and chlorophylls) identified in pansy flowers petals
